# Near-infrared spectroscopy (NIRS) neurofeedback as a treatment for children with attention deficit hyperactivity disorder (ADHD)—a pilot study

**DOI:** 10.3389/fnhum.2014.01038

**Published:** 2015-01-07

**Authors:** Anna-Maria Marx, Ann-Christine Ehlis, Adrian Furdea, Martin Holtmann, Tobias Banaschewski, Daniel Brandeis, Aribert Rothenberger, Holger Gevensleben, Christine M. Freitag, Yvonne Fuchsenberger, Andreas J. Fallgatter, Ute Strehl

**Affiliations:** ^1^Institute for Medical Psychology and Behavioral Neurobiology, University of TuebingenTuebingen, Germany; ^2^Department of Psychiatry and Psychotherapy, Psychophysiology and Optical Imaging, University of TuebingenTuebingen, Germany; ^3^LWL-University Hospital for Child and Adolescent Psychiatry, Ruhr-University BochumHamm, Germany; ^4^Department of Child and Adolescent Psychiatry and Psychotherapy, Central Institute of Mental Health, Medical Faculty Mannheim of the University of HeidelbergMannheim, Germany; ^5^Department of Child and Adolescent Psychiatry, University of ZuerichZuerich, Switzerland; ^6^Clinic for Child and Adolescent Psychiatry, University Medical Center of GoettingenGoettingen, Germany; ^7^Department of Child and Adolescent Psychiatry, Psychosomatics and Psychotherapy, Goethe-University Frankfurt am MainFrankfurt am Main, Germany

**Keywords:** near-infrared spectroscopy (NIRS), fNIRS, neurofeedback, attention deficit hyperactivity disorder (ADHD), children, prefrontal cortex (PFC)

## Abstract

In this pilot study near-infrared spectroscopy (NIRS) neurofeedback was investigated as a new method for the treatment of Attention Deficit-/Hyperactivity Disorder (ADHD). Oxygenated hemoglobin in the prefrontal cortex of children with ADHD was measured and fed back. 12 sessions of NIRS-neurofeedback were compared to the intermediate outcome after 12 sessions of EEG-neurofeedback (slow cortical potentials, SCP) and 12 sessions of EMG-feedback (muscular activity of left and right musculus supraspinatus). The task was either to increase or decrease hemodynamic activity in the prefrontal cortex (NIRS), to produce positive or negative shifts of SCP (EEG) or to increase or decrease muscular activity (EMG). In each group nine children with ADHD, aged 7–10 years, took part. Changes in parents’ ratings of ADHD symptoms were assessed before and after the 12 sessions and compared within and between groups. For the NIRS-group additional teachers’ ratings of ADHD symptoms, parents’ and teachers’ ratings of associated behavioral symptoms, childrens’ self reports on quality of life and a computer based attention task were conducted before, 4 weeks and 6 months after training. As primary outcome, ADHD symptoms decreased significantly 4 weeks and 6 months after the NIRS training, according to parents’ ratings. In teachers’ ratings of ADHD symptoms there was a significant reduction 4 weeks after the training. The performance in the computer based attention test improved significantly. Within-group comparisons after 12 sessions of NIRS-, EEG- and EMG-training revealed a significant reduction in ADHD symptoms in the NIRS-group and a trend for EEG- and EMG-groups. No significant differences for symptom reduction were found between the groups. Despite the limitations of small groups and the comparison of a completed with two uncompleted interventions, the results of this pilot study are promising. NIRS-neurofeedback could be a time-effective treatment for ADHD and an interesting new option to consider in the treatment of ADHD.

## Introduction

Attention Deficit-/Hyperactivity Disorder (ADHD) is characterized by the main symptoms of inattention, hyperactivity and impulsivity, leading to deficits in social and/or academic functioning.

In the model of prefrontal lobe executive functions according to Barkley (1997), a deficit in behavioral inhibition in ADHD leads to deficits in executive functions, such as working memory, and in consequence to a deficient self control. Increasing behavioral inhibition should in consequence lead to an increased self control and symptom reduction. Deficits in executive functioning can be observed in children with ADHD compared to healthy controls (Martinussen et al., 2005; Willcutt et al., 2005). On a neurophysiological level, central nervous hypo-arousal during working memory tasks measured with fMRI was found in children with ADHD compared to healthy controls (Dickstein et al., 2006; Paloyelis et al., 2007), as well as alterations in the prefrontal cortex (Brennan and Arnsten, 2008) (see also NIRS studies below).

Neurofeedback as a treatment for ADHD can be interpreted as a way to increase behavioral inhibition. Neurofeedback is commonly EEG-feedback of frequency bands or slow cortical potentials (SCP), measuring and feeding back electrical brain activity (Arns et al., 2013; Holtmann et al., 2014b). The training protocols are based on findings of hypoarousal in the resting state EEG (Barry et al., 2003a) or findings on divergent event-related potentials (Barry et al., 2003b). EEG-neurofeedback has been proven to be an effective treatment for ADHD as regards to the reduction of inattention, impulsivity and hyperactivity (Arns et al., 2009).

An alternative method to assess brain activity is functional near-infrared spectroscopy (NIRS), measuring hemodynamic correlates of neural activity. Light in the near-infrared spectrum is absorbed to different amounts by oxygenated and deoxygenated hemoglobin allowing to determine relative concentration changes on the cortical surface (Fallgatter and Strik, 1997; Obrig et al., 2000; for an overview on applications see: Ehlis et al., 2014). Higher brain activity is thereby reflected by concentration increases (decreases) of oxygenated (deoxygenated) hemoglobin.

In most of the few NIRS studies comparing children with ADHD to healthy controls in different executive functioning tasks, altered prefrontal activity was observed: some reported reduced activity in ADHD (Negoro et al., 2010; Inoue et al., 2012; Xiao et al., 2012), some reported increased activity in ADHD (Weber et al., 2005; Jourdan Moser et al., 2009). While a few studies suggest a more pronounced involvement of the right lateral prefrontal cortex (Xiao et al., 2012; Yasumura et al., 2014), most report no specific lateralization or even clear bilateral deficits (e.g., Ehlis et al., 2008; Negoro et al., 2010; Inoue et al., 2012). Based on these findings, neurofeedback of hemodynamic activity in the prefrontal cortex could lead to a more effective use of cognitive resources, similar to EEG-neurofeedback.

fMRI-neurofeedback of hemodynamic activity has been investigated in healthy adults, showing the possibility of acquiring self-regulation rapidly in only three to four sessions (Weiskopf et al., 2003, 2004; Caria et al., 2007, 2010). The same was observed for NIRS-neurofeedback in healthy adults (Ayaz et al., 2009). In comparison to EEG-neurofeedback requiring around 30 sessions to gain sufficient self-control, NIRS-neurofeedback could be an interesting alternative, possibly allowing changes in symptomatology in fewer sessions of feedback.

Based on these findings we wanted to investigate NIRS-neurofeedback as a new method of neurofeedback for children with ADHD, aimed at gaining control over prefrontal hemodynamics. Based on the above mentioned findings in fMRI- and NIRS-studies, the left and right dorsolateral prefrontal cortex was chosen as region of interest for the neurofeedback signal, representing also a key region of executive functioning. Concentration changes in oxygenated hemoglobin were used as feedback signal due to several (partly interrelated) reasons: First, oxygenated hemoglobin was found to show the strongest correlation with the fMRI BOLD signal, probably because of its superior signal-to-noise ratio as compared to deoxygenated hemoglobin (Strangman et al., 2002). Second, depending on the vascular characteristics of the brain tissue covered by the NIRS optodes, the signal course of deoxygenated hemoglobin can show considerable differences, with cortical activation leading to (the usually expected) decreases, increases or even no changes in HHb concentration. Oxygenated hemoglobin, on the other hand, consistently shows concentration increases during active task periods (Yamamoto and Kato, 2002) allowing for a more reliable interpretation of oxy-Hb data. Third, previous findings also suggest that the amplitude of change is always larger for oxygenated than for deoxygenated hemoglobin (Yamamoto and Kato, 2002), which is a critical point in feedback trainings that rely on single-trial NIRS data (as in our case).

Besides the general aim to investigate the feasibility of NIRS-neurofeedback especially for children with ADHD, the study was designed to assess as primary outcome if NIRS-neurofeedback leads to a reduction of ADHD in parents’ ratings and if changes persist 6 months after the training. Additionally, decreased teachers’ ratings of ADHD symptoms, decreased parents’ and teachers’ ratings of associated behavioral symptoms, improvements in children’s self-rated quality of life and in the performance in a computer based attention task were expected. As an active control condition neurofeedback of SCPs was selected, as a semi-active control condition feedback of muscular activity of the left and right musculus supraspinatus was chosen (for an overview of control conditions in neurofeedback see Arns et al., 2013). We expected comparable changes in symptomatology compared to EEG-neurofeedback and greater changes in comparison to EMG-feedback after 12 sessions of training.

Additionally, the hemodynamic brain activity was measured during the NIRS-neurofeedback training sessions and a working memory task with parallel NIRS measurement was conducted to measure changes in prefrontal brain activity before and after the training. The hemodynamic data are not part of this paper and will be published separately.

The study was approved by the Ethics Committee of the Medical Faculty of the University of Tuebingen and conducted according to the ethical guidelines and principles of the international Declaration of Helsinki. The multicentre study (ISRCTN76187185) was approved by all local Ethics Committees according to the Declaration of Helsinki. Written informed consent was obtained from parents and children.

## Materials and methods

### Participants

Inclusion criteria were age between 7;0 and 10;11 years and a full-scale intelligence quotient over 80 (percentile >9, assessed with the Colored Progressive Matrices CPM, Raven et al., 1998; German version: Bulheller and Häcker, 2006) and a pre-diagnosis of ADHD by a child psychiatrist, pediatrician or clinical psychologist. Exclusion criteria were an intelligence level under 80 (percentile ≤9), medical or neurological disorders, psychiatric disorders other than oppositional defiant disorder and current participation in a psychotherapeutic treatment.

27 children with ADHD combined type (age *M* = 8.90 years, *sd* = 1.02; 9 female) participated in the study. The diagnosis was confirmed with the supplement for ADHD (German version: Delmo et al., 2000) of the semi-structured interview Kiddie-Sads-Present and Lifetime Version (Kaufman et al., 1997; Kaufman and Schweder, 2003), using DSM-IV criteria.

Nine children (3 female) with a mean age of *M* = 9.00 years (*sd* = 1.26) took part in the NIRS-feedback. For the EEG- and the EMG-group, 18 children were matched to the NIRS-group for gender, medication status and age (EEG-group: *M* = 8.85 years, *sd* = 0.99, 3 female; EMG-group: *M* = 8.83 years, *sd* = 0.88, 3 female). The children of these two groups were participants in a multicenter neurofeedback study (ISRCTN76187185, Holtmann et al., 2014a) with a total of 144 participants recruited with identical inclusion and exclusion criteria.

As additional screening instruments, all parents rated the child’s behavior on the Child behavior checklist (CBCL, Achenbach, 1991; German version: Arbeitsgruppe Deutsche Child Behavior Checklist, 1998) and their own parenting behavior on the Parenting Scale (Arnold et al., 1993; German short version EFB-K: Miller, 2001). Medication status was assessed; seven children in each group with a medication of methylphenidate stopped medication at least 48 h before the pretest, the post test 2 and the follow-up test. There were no other medication agents (amphetamine, atomoxetine) prescribed in the NIRS-sample, so the matching included only children with a medication of methylphenidate. The groups did not differ significantly in age, IQ percentile, total score of parenting behavior and total score of child’s behavior (see Table 1).

**Table 1:** **Description of the sample**.

		NIRS-group *n* = 9	EEG-group *n* = 9	EMG-group *n* = 9	Kruskal-Wallis
Age	*Mdn*	8.92	9.17	8.83	*H*(2) = 0.28
	*IQR*	7.67–10.25	8.00–9.83	8.25–9.50	*p* = 0.869
CPM Percentile	*Mdn*	76.00	69.00	85.00	*H*(2) = 1.94
	*IQR*	61.50–97.50	32.00–90.00	72.50–93.50	*p* = 0.380
EFB-K total Score	*Mdn*	2.77	3.00	3.20	*H*(2) = 0.78
	*IQR*	2.23–3.56	2.75–3.50	2.70–3.30	*p* = 0.678
CBCL total Raw score	*Mdn*	47.00	36.00	35.00	*H*(2) = 3.03
	*IQR*	35.00–66.00	26.00–56.50	24.50–52.50	*p* = 0.220

*Mdn = median, IQR = interquartile range, CPM = Colored Progressive Matrices, EFB-K = Parenting scale, CBCL = Child Behavior Checklist*.

### Procedure and measurement instruments

The nine children in the NIRS-group were recruited in the same manner as the children of the two other groups through local advertisements and through pediatricians and child psychiatrists. For the matching of EEG- and EMG-group, 80 complete datasets were used and only gender, medication status, age and IQ were transferred. After matching, the complete dataset was provided. Main matching criteria were same gender and same medication status, followed by nearest age and nearest IQ. The NIRS-group received 12 sessions, the EEG- and EMG-group received 25 sessions of training. For the inter-group comparison, the FBB-ADHS was used after 12 sessions (see Table 2). As there are no NIRS-neurofeedback studies investigating children with ADHD so far, the amount of 12 sessions for the NIRS-group was based on the findings in fMRI- and NIRS-neurofeedback with healthy subjects using three to four sessions (see Introduction) and was adapted for practical reasons to the intermediate outcome of the multicentric study, to allow group comparison. The outcome of the full 12 session NIRS-training was thus compared to the intermediate 12 session outcome of the longer EEG- and EMG-training, in order to test for a more rapid clinical improvement with NIRS-neurofeedback.

**Table 2:** **Measurement points and instruments for within- and between-group comparisons**.

Measurement point	Measurement instruments		
	Parents	Teachers	Children
Pretest	**FBB-ADHS** SDQ	FBB-ADHS SDQ	KID-KINDL TAP 2.2 Go/NoGo and Flexibility
Post Test 1 (after session 12)	**FBB-ADHS**
Post Test 2 (4 weeks after session 12)	FBB-ADHS SDQ	FBB-ADHS SDQ	KID-KINDL TAP 2.2 Go/NoGo and Flexibility
Follow up Test (6 months after session 12)	FBB-ADHS SDQ	FBB-ADHS SDQ	KID-KINDL TAP 2.2 Go/NoGo and Flexibility

*Bold marked FBB-ADHS = measurements in all three groups for group comparison. FBB-ADHS = Rating scale for ADHD, SDQ = Strengths and Difficulties Questionnaire, KID-KINDL = Questionnaire for health-related quality of life, TAP 2.2 = Test Battery for Attentional Performance*.

For the pre-post comparison of the NIRS-group ADHD symptoms rated by parents and teachers in the Rating Scale for Attention Deficit Hyperactivity Disorder (Fremdbeurteilungsbogen für Aufmerksamkeitsdefizit-/Hyperaktivitätsstörung, FBB-ADHS) were assessed as main dependent variables. The parent-rated FBB-ADHS was the primary outcome. The FBB-ADHS is part of the Diagnostic System for Mental Disorders in Childhood and Adolescence (DISYPS-II, Döpfner et al., 2008). The FBB-ADHS covers the diagnostic criteria for the combined type of ADHD according to the Diagnostic and Statistical Manual of the American Psychiatric Association (4th edition; DSM-IV, American Psychiatric Association, 2000) and can be regarded as the German equivalent of the SNAP-IV. Associated behavioral symptoms were assessed with the Strengths and Difficulties Questionnaire (SDQ, Goodman, 1997; German Version: Rothenberger and Woerner, 2004), rated by parents and teachers. Children rated their quality of life in the Kindl-Questionnaire for health-related quality of life (KID-KINDL, Ravens-Sieberer, 2003) and childrens’ attention and impulsivity were measured with two subtests (Flexibility, Go/NoGo) of a computer based attention task (Test Battery for Attentional Performance, TAP, Zimmermann and Fimm, 2009). The TAP subtest Go/NoGo measures the ability to inhibit reactions (performance variables: median of reaction times, standard deviation of reaction times, omissions and commissions); the subtest Flexibility measures the ability to change the focus of attention (performance variables: median of reaction times, standard deviation of reaction times, commissions). All instruments were applied before training (pretest), 4 weeks (post test 2) and 6 months (follow-up test) after the training. Table 2 gives an overview of measurement points and instruments.

The childrens’ pediatricians or the childrens’ psychiatrists were asked to rate severity of psychopathology and improvement after treatment on the Clinical Global Impression Scale (Guy, 1976). This data was not analyzed and will not be reported due to low return rates (pretest *n* = 6, post test *n* = 3, follow up *n* = 2).

### NIRS-neurofeedback

NIRS-neurofeedback training consisted of twelve sessions within 4–6 weeks, with 2–3 sessions per week. Each session comprised three blocks of NIRS-neurofeedback. After the 12 sessions, children were instructed to practice their strategy for 3 weeks in attention-requiring situations at home or school, to facilitate the transfer to everyday life. In order to motivate the children, the whole training was accompanied by a token system in which the children could gain points and swap them for small toys. Tokens were given for good cooperation during a training session, independent of achievement.

**Figure 1:**
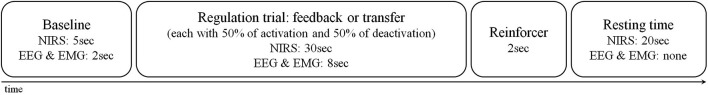
**Trial design of NIRS-, EEG- and EMG- blocks**. One NIRS session consisted of 2 feedback blocks each with 12 trials and 1 transfer block with 8 trials. An EEG and EMG session consisted of 3 feedback blocks and 1 transfer block each with 40 trials.

The basic parameters of the three trainings (NIRS, EEG, EMG) were comparable. Each session lasted approximately 1 hour with 32 min effective feedback time. The visual layout of the feedback was identical. Compared to electrical brain and muscular activity, changes in hemodynamic activity are somewhat delayed and need more time to return back to baseline. In consequence, hemodynamic neurofeedback trials were designed with longer regulation and resting times (see Figure 1 for a comparison of the three training protocols).

One session comprised 2 feedback blocks each lasting 12 min and one transfer block lasting 8 min. A feedback block consisted of 12 regulation trials. One trial consisted of 20 s resting time, 5 s baseline measurement and 30 s regulation time (see Figure 1 for trial design). The task was to increase or decrease the hemodynamic activity in the prefrontal cortex (in 50% of cases activation, in 50% deactivation, in a random order). As feedback the children saw an object on a screen (e.g., a fish), moving from left to right and depicting concentration changes in oxygenated hemoglobin. An arrow in the middle of the screen indicated if activation (pointing upwards) or deactivation (pointing downwards) was expected. In activation trials the concentration of oxygenated hemoglobin should increase in comparison to the baseline, in deactivation trials it should decrease. At the end of a successful trial (= the object was flying at least 7 s of the last 15 s regulation time in the expected direction) a sun was shown on the screen as a visual reinforcer. A transfer block consisted of 8 regulation trials in which the moving feedback object was not shown, but the sun at the end of the trial indicated whether the participant was successful. The transfer blocks were included in order to facilitate the transfer to everyday life.

**Figure 2:**
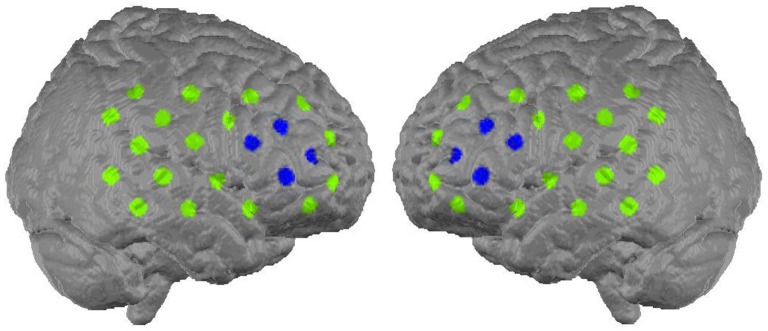
**Alignment of the 44 NIRS channels on the cortex surface (Marx, 2014).** The eight channels from which the feedback signal was computed are marked with blue (figure buildt with MATLAB based on MNI coordinates, available: http://www.jichi.ac.jp/brainlab/virtual_registration/Result3x5_E.html, Tzourio-Mazoyer et al., 2002; Tsuzuki et al., 2007).

The neurofeedback signal reflected relative concentration changes of oxygenated hemoglobin in the prefrontal cortex. A 52-channel NIRS system (Hitachi Optical Topography System ETG-4000) was placed over frontal and temporal areas and linked to a neurofeedback device (NeuroConn THERA-PRAX). For the measurement 46 optodes (44 NIRS channels) were used, arranged on two 3 × 5 probesets. The probesets were oriented along positions of the 10–20-system of electrode placement. The lowest row of both probesets was oriented frontally with Fpz as midpoint, while the second optode from occipital in the lowest row on each side was lying on T3 respectively T4 (see Figure 2 for channel positions). The neurofeedback signal, that is the signal that controls the “flying” object on the computer’s screen, was based on mean concentration changes in oxygenated hemoglobin measured over the right and left dorsolateral prefrontal cortex and computed using the following procedure: In a first step, for each sample in time, the average of the signals from four NIRS channels located over the left and right dorsolateral prefrontal cortex (see Figure 2, blue marking) was computed. This was followed by subtracting the average of the particular probeset (22 channels) per side (common average reference). In a last step, the resulting two signals (one corresponding to each side) were averaged and used to provide feedback. This method was adopted to minimize the effect of hemodynamic artifacts induced by breathing, head movements or skin blood flow.

The NIRS signals were transmitted to a personal computer via TCP/IP protocol for further processing. The feedback signal was computed online using a self-programmed MATLAB routine and it served as input signal for the neurofeedback device.

### EEG-neurofeedback and EMG-feedback

The EEG- and EMG-group participated in 25 sessions, with a 3 week practice break including an intermediate outcome assessment using the FBB-ADHS after 12 sessions. One session consisted of 3 feedback blocks (with the same visualization as in the NIRS-group) and one transfer block (without feedback object) each lasting 8 min. The blocks consisted of 40 regulation trials. One regulation trial comprised 2 s baseline measurement and 8 s regulation time (50% of cases activation, 50% deactivation, in a random order, see Figure 1 for trial design, a detailed description is provided in Holtmann et al., 2014a).

The feedback was conducted with the NeuroConn NEURO-PRAX (identical software to THERA-PRAX, possibility to measure more EEG channels). Nine Ag/AgCl ring electrodes were used, one at Cz, two at the right and left mastoid (A1 and A2), two central over and under the left eye, two at the left and right corner of both eyes and two at the right and left musculus supraspinatus above the shoulders.

In the EEG-group the EEG-signal (slow cortical potentials at Cz referenced against A1, online corrected for eye movements, ground electrode at A2) was fed back, in the EMG-group the EMG-signal was fed back. The task in the EEG-group was to produce a positive or negative shift of the SCPs in comparison to the baseline. The task in the EMG-group was to increase muscle tension on the left side while decreasing it on the right side and vice versa in comparison to the baseline. At the end of a successful trial (= the object was flying at least 2 s of the last 4 s regulation time in the expected direction) a sun was shown on the screen as a visual reinforcer.

### Data analysis and statistics

For the FBB-ADHS, the SDQ and the KID-KINDL total scores were calculated according to the test instructions. A higher score in the FBB-ADHS implies more severe ADHD symptoms and a higher score in the SDQ implies a higher occurrence of associated behavioral symptoms, including hyperactivity. A higher score in the KID-KINDL implies a higher self-rated quality of life. For the two TAP subtests, medians of reaction times, standard deviations of reaction times and the numbers of commissions and omissions (only Go/NoGo) were assessed and analyzed. Higher medians of reaction times represent slower reactions; higher standard deviations of reaction times represent a higher variability of reaction times.

IBM SPSS Version 20 was used for statistical analysis. Due to small sample-size, non-parametric tests were applied. Significance level was set to α ≤ 0.05. Friedman’s ANOVAS were conducted for comparisons within the NIRS-group (pretest, post test 2, follow-up test) for the dependent variables. For *post hoc* analysis Wilcoxon signed-rank tests were conducted. Additionally, effect sizes were calculated $\left(r=\frac{z}{\sqrt{N}}\right)$.

For the comparison of the three groups the initial values of parents’ and teachers’ ratings of the FBB-ADHD and the SDQ, the child-rated KID-KINDL and the performance data of the TAP subtests were compared with Kruskal-Wallis-Tests and *post hoc* Mann-Whitney *U* Tests, to ensure the general comparability of the three groups. To assess the pre-post effects of the twelve training sessions for each group separately, three Wilcoxon signed rank tests were conducted for the total scores of the FBB-ADHS comparing pretest and post test 1 within each group. Additionally, differences of the scores were calculated for each group (total score at post test 1 minus total score at pretest, a higher difference implies a higher symptom reduction), and these differences were compared between groups in a Kruskal-Wallis-Test.

## Results

### Within-group comparisons for the NIRS-group

For an overview of all medians and interquartile ranges for the dependent variables of the NIRS-group at all measurement points with test statistics see Table 3. Five teacher ratings were not included in the data analysis: one was not sent back (SDQ + FBB-ADHS), one was returned empty (SDQ + FBB-ADHS), three could not be assigned to a measurement point (2 SDQ + FBB-ADHS, 1 SDQ).

**Table 3:** **Medians and interquartile ranges for the dependent variables of the NIRS-group at all measurement points with test statistics**.

		Pretest	Post test 2	Follow-up test	Friedman’s ANOVAS
FBB-ADHS total score parents	*Mdn*	1.65	1.05	1.05	*χ*^2^(2) = 6.59
	*IQR*	1.33–2.15	0.68–1.33	0.88–1.25	***p* = 0.037**
FBB- ADHS total score teachers	*Mdn*	1.10 (*n* = 7)	1.00 (*n* = 8)	1.03 (*n* = 8)	*χ*^2^(2) = 6.33
	*IQR*	0.80–2.15	0.44–2.05	0.66–1.51	***p* = 0.042**
KID-KINDL total score	*Mdn*	4.13	4.46	4.17	*χ*^2^(2) = 2.00
	*IQR*	3.75–4.46	3.40–4.71	3.98–4.40	*p* = 0.368
SDQ total score parents	*Mdn*	18.00	16.00	14.00	*χ*^2^(2) = 5.88
	*IQR*	16.50–23.00	11.50–19.50	9.50–17.50	*p* = 0.053
SDQ total score teachers	*Mdn*	13.00 (*n* = 7)	10.00 (*n* = 7)	9.50 (*n* = 8)	*χ*^2^(2) = 2.78
	*IQR*	10.00–18.00	6.00–27.00	7.50–23.75	*p* = 0.249
TAP Go/NoGo median reaction time	*Mdn*	551.00	580.00	497.00	*χ*^2^(2) = 6.91
	*IQR*	450.50–600.00	470–606.50	440.00–520.00	***p* = 0.032**
TAP Go/NoGo standard deviation reaction time	*Mdn*	150.00	122.00	89.00	*χ*^2^(2) = 8.97
	*IQR*	112.50–185.50	87.00–149.50	79.00–113.50	***p* = 0.011**
TAP Go/NoGo commissions	*Mdn*	4.00	0.00	0.00	*χ*^2^(2) = 12.96
	*IQR*	1.50–11.50	0.00–2.00	0.00–3.50	***p* = 0.002**
TAP Go/NoGo omissions	*Mdn*	0.00	0.00	0.00	*χ*^2^(2) = 5.38
	*IQR*	0.00–2.00	0.00–0.00	0.00–0.50	*p* = 0.068
TAP Flexibility median reaction time	*Mdn*	1276.00	929.00	1012.00	*χ*^2^(2) = 6.22
	*IQR*	843.00–1468.00	776.50–1092.50	719.50–1144.50	***p* = 0.045**
TAP Flexibility standard deviation reaction time	*Mdn*	534.00	350.00	320.00	*χ*^2^(2) = 6.22
	*IQR*	272.00–630.00	285.00–409.00	227.00–384.50	***p* = 0.045**
TAP Flexibility commissions	*Mdn*	12.00	9.00	5.00	*χ*^2^(2) = 4.22
	*IQR*	6.00–21.00	4.00–17.00	3.50–8.50	*p* = 0.121

*Reaction times in milliseconds, Mdn = Median, IQR = interquartile range, bold = significant at α ≤ 0.05, FBB-ADHS = Rating scale for ADHD, SDQ = Strengths and Difficulties Questionnaire, KID-KINDL = Questionnaire for health-related quality of life, TAP = Test Battery for Attentional Performance; if not reported otherwise: n = 9*.

#### FBB-ADHS

**Figure 3:**
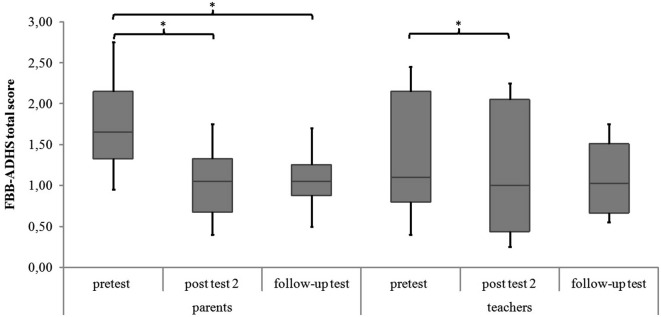
**FBB-ADHS total score of parents’ and teachers’ ratings for all meeasurement points**. Middle line of boxes = median, box = interquartile range, error bars = minimum respectively maximum, * = significant.

There are significant differences in the measurement points of the FBB-ADHS total score for the NIRS-group in parents’ and teachers’ ratings (parents: χ^2^(2) = 6.59, *p* = 0.037; teachers: χ^2^(2) = 6.33, *p* = 0.042). In the *post hoc* analysis of parents’ ratings, ADHD symptoms significantly decreased from pretest to post test 2 (*z* = −2.49, *p* = 0.013, *r* = −0.587) as well as from pretest to follow-up test (*z* = −2.31, *p* = 0.021, *r* = −0.544). There was no significant difference between parents’ ratings from post test 2 to follow-up test (*z* = −0.51, *p* = 0.611, *r* = −0.120). The *post hoc* analysis of teachers’ ratings revealed a significant decrease of ADHD symptoms in teachers’ ratings from pretest to post test 2 (*z* = −2.21, *p* = 0.027, *r* = −0.535), but not from pretest to follow-up test (*z* = −1.69, *p* = 0.091, *r* = −0.410) or from post test 2 to follow-up test (*z* = −0.34, *p* = 0.735, *r* = −0.080). For medians and interquartile ranges of FBB-ADHS total scores see Figure 3 and Table 3.

#### SDQ and KID-KINDL

**Figure 4:**
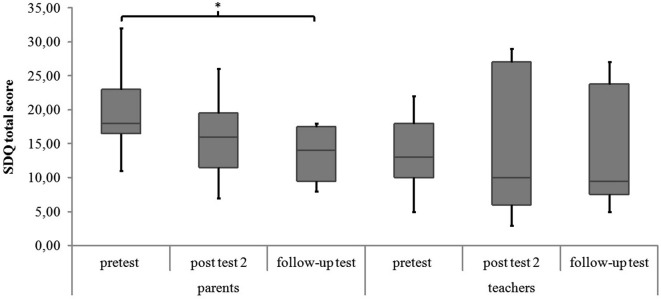
**SDQ total score of parents’ and teachers’ ratings for all measurement points**. Middle line of boxes = median, box = interquartile range, error bars = minimum respectively maximum, * = significant.

There were no significant differences in parents’ and teachers’ ratings of associated behavioral symptoms (SDQ total scores) and in childrens’ ratings of quality of life (KID-KINDL) for the three measurement points (parents’ ratings SDQ: χ^2^(2) = 5.88, *p* = 0.053; teachers’ ratings SDQ: χ^2^(2) = 2.78, *p* = 0.249; childrens’ ratings KID-KINDL: χ^2^(2) = 2.00, *p* = 0.368). In the *post hoc* analysis there was a significant decrease in parents’ ratings of the SDQ total score from pretest to follow-up test (*z* = −2.55, *p* = 0.011, *r* = −0.602). See Figure 4 and Table 3 for medians and interquartile ranges of the SDQ total scores.

#### TAP

##### Go/NoGo

**Figure 5:**
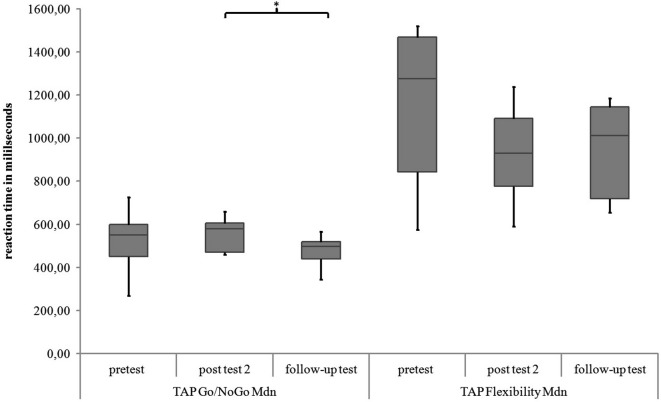
**Medians of reaction times for the TAP subtests Go/NoGo and Flexibility for all measurement points**. Middle line of boxes = median, box = interquartile range, error bars = minimum respectively maximum, * = significant.

**Figure 6:**
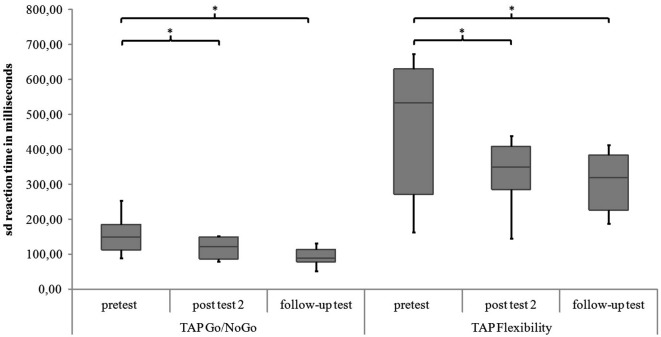
**Standard deviations of reaction times for the TAP subtests Go/NoGo and Flexibility for all measurement points**. Middle line of boxes = median, box = interquartile range, error bars = minimum respectively maximum, * = significant.

**Figure 7:**
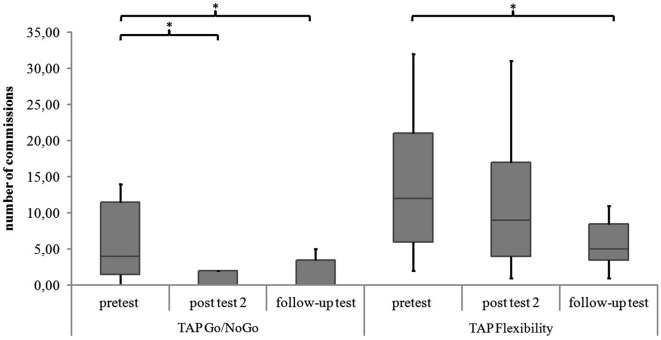
**Commissions in the TAP subtests Go/NoGo and Flexibility for all measurement points**. Middle line of boxes = median, box = interquartile range, error bars = minimum respectively maximum, * = significant.

For the subtest Go/NoGo significant differences were found for the three measurement points in standard deviations of reaction times (χ^2^(2) = 8.97, *p* = 0.011), commission errors (χ^2^(2) = 12.96, *p* = 0.002) and medians of reaction times (χ^2^(2) = 6.91, *p* = 0.032). In the *post hoc* analysis there was a significant difference in the medians of reaction times from post test 2 to follow-up test (*z* = −2.52, *p* = 0.012, *r* = −0.595), as shown in Figure 5. The children reacted faster 6 months after the training than 4 weeks after the training. In the *post hoc* analyses of the standard deviations of reaction times there was a significant difference between pretest and post test 2 (*z* = −2.38, *p* = 0.017, *r* = −0.561) as well as between pretest and follow up test (*z* = −2.55, *p* = 0.011, *r* = −0.600, see Figure 6). The children reacted with less variability 4 weeks and 6 months after the training in comparison to prior to the training. There were significant differences in commission errors from pretest to *post test* 2 (*z* = −2.53, *p* = 0.012, *r* = −0.596) and from pretest to follow-up test (*z* = −2.37, *p* = 0.018, *r* = −0.559), as shown in Figure 7. The children made fewer commission errors after the training.

##### Flexibility

In the subtest Flexibility there were significant differences for the medians of reaction times (χ^2^(2) = 6.22, *p* = 0.045) and the standard deviations of reaction times (χ^2^(2) = 6.22, *p* = 0.045). In the *post hoc* analysis there were no significant differences for the medians of reaction times (see Figure 5). In the *post hoc* analysis of the standard deviations of reaction times there was a significant decrease of variability from pretest to post test 2 (*z* = −2.31, *p* = 0.021, *r* = −0.544) and from pretest to follow-up test (*z* = −2.31, *p* = 0.021, *r* = −0.544), see Figure 6. There was also a significant decrease of commission errors from pretest to follow-up test (*z* = −1.96, *p* = 0.050, *r* = −0.462), see Figure 7.

### Within and between group comparisons for EEG-, EMG- and NIRS-group

There were no significant differences between groups in the pretest values of parents’ and teachers’ rating of the ADHD symptoms in the FBB-ADHS and in the teachers’ rating of associated behavioral symptoms in the SDQ (see Table 4). Childrens’ ratings of the quality of life in the KID-KINDL and parents’ ratings of associated behavioral symptoms in the SDQ differed significantly between groups (see Table 4). *Post hoc* analyses for the SDQ revealed a significant higher score in the EMG-group in comparison to the NIRS-group (NIRS vs. EEG: *U* = 21.50, *z* = −1.68, *p* = 0.094; NIRS vs. EMG: *U* = 15.00, *z* = −2.26, *p* = 0.024; EEG vs. EMG: *U* = 26.50, *z* = −1.24, *p* = 0.222). *Post hoc* analyses for the KID-KINDL showed significant higher quality of life scores in the NIRS-group in comparison to the EEG-group (*U* = 0.00, *z* = −3.58, *p* = 0.000) as well as in comparison to the EMG-group (*U* = 0.00, *z* = −3.58, *p* = 0.000), there was no difference between EEG- and EMG-group (*U* = 26.50, *z* = −1.25, *p* = 0.222).

**Table 4:** **Medians and interquartile ranges of FBB-ADHS, KID-KINDL and SDQ at pretest with group statistics**.

		NIRS-group *n* = 9	EEG-group *n* = 9	EMG-group *n* = 9	Kruskal-Wallis
FBB-ADHS parents total score	*Mdn*	1.65	2.00	1.75	*H*(2) = 2.66
	*IQR*	1.33–2.15	1.75–2.23	1.60–1.90	*p* = 0.264
FBB-ADHS teachers total score	*Mdn*	1.10 (*n* = 7)	1.20 (*n* = 8)	1.95 (*n* = 8)	*H*(2) = 1.73
	*IQR*	0.80–2.15	0.61–1.45	0.56–2.45	*p* = 0.421
KID-KINDL total score	*Mdn*	4.13	2.88	3.08	*H*(2) = 18.12
	*IQR*	3.75–4.46	2.61–2.96	2.59–3.11	***p* = 0.000**
SDQ parents total score	*Mdn*	18.00	23.00	24.00	*H*(2) = 6.33
	*IQR*	16.50–23.00	19.50–28.50	21.00–32.00	***p* = 0.042**
SDQ teachers total score	*Mdn*	13.00 (*n* = 7)	17.00 (*n* = 8)	23.00 (*n* = 8)	*H*(2) = 3.41
	*IQR*	10.00–18.00	13.00–18.50	12.50–28.50	*p* = 0.182

*Reaction times in milliseconds, Mdn = median, IQR = interquartile range, bold = significant at α ≤ 0.05, FBB-ADHS = Rating scale for ADHD, SDQ = Strengths and Difficulties Questionnaire, KID-KINDL = Questionnaire for health-related quality of life; if not reported otherwise: n = 9*.

**Figure 8:**
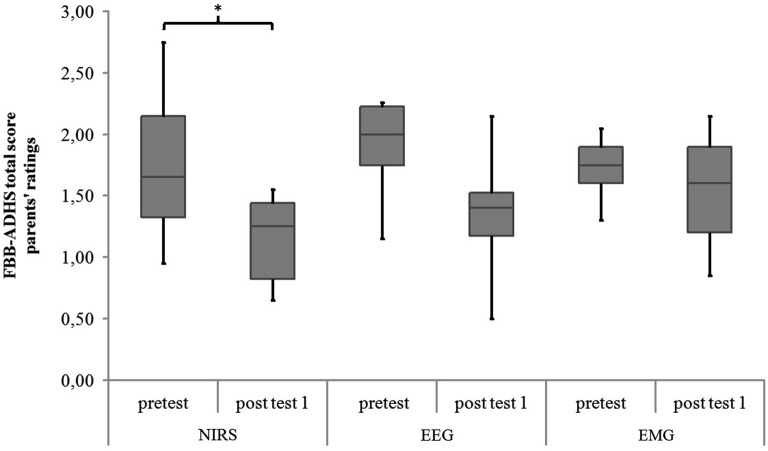
**FBB-ADHS total score of parents’ ratings for NIRS-, EEG- and EMG-group at pretest and post test 1**. Middle line of boxes = median, box = interquartile range, error bars = minimum respectively maximum, * = significant.

Within-group comparisons to assess the pre-post effect of the twelve sessions separately for each group revealed a significant difference in the parents’ rating of the FBB-ADHS only for the NIRS-group (*z* = −2.25, *p* = 0.024,* r* = −0.531, see Table 5 and Figure 8). A trend for a lower FBB-ADHS score was observed for the EEG-group (*z* = −1.90, *p* = 0.058, *r* = −0.447) and the EMG-group (*z* = −1.84, *p* = 0.066, *r* = −0.434).

**Table 5:** **Parents’ ratings of the FBB-ADHS at pretest and post test 1 for the three groups with test statistics**.

		FBB-ADHS total score pretest	FBB-ADHS total score post test 1	Wilcoxon signed rank Test
NIRS-group	*Mdn*	1.65	1.25	*z* = −2.25
	*IQR*	1.33–2.15	0.83–1.44	***p* = 0.024**
				*r* = −0.531
EEG-group	*Mdn*	2.00	1.40	*z* = −1.90
	*IQR*	1.75–2.23	1.18–1.53	*p* = 0.058
				*r* = −0.447
EMG-group	*Mdn*	1.75	1.60	*z* = −1.84
	*IQR*	1.60–1.90	1.20–1.90	*p* = 0.066
				*r* = −0.434

Mdn = median, IQR = interquartile range, r = effect size, bold = significant at α ≤ 0.05, FBB-ADHS = Rating scale for ADHD.

Comparing the three groups in the differences of parents’ ratings of the FBB-ADHS (post test 1 values minus pretest values), there were no significant differences between the groups (NIRS: *Mdn* = −0.65, *IQR* = −1.03 − −0.11; EEG: *Mdn* = −0.60, *IQR* = −1.06 – 0.03; EMG: *Mdn* = −0.20, *IQR* = −0.38–0.04; *H*(2) = 2.72, *p* = 0.256).

## Discussion

In this pilot study NIRS-neurofeedback as a new method of neurofeedback training for children with ADHD was investigated. Hemodynamic brain activity in the dorsolateral prefrontal cortex was measured and fed back. Children should learn to gain control over their brain activity in 12 training sessions and 3 weeks of transfer exercises. Primary outcome was the effect on ADHD symptoms rated by parents. Teachers’ ratings of symptoms as well as ratings of associated behavioral symptoms by parents and teachers, self-rated quality of life and performance in a computer based attention task were assessed. In addition, a comparison with two other feedback methods (EEG, EMG) was carried out.

### NIRS -neurofeedback—effects and feasibility

As primary outcome, parents’ ratings of ADHD symptoms in the NIRS-group were significantly reduced 4 weeks and 6 months after the training. Teachers’ ratings of ADHD symptoms showed a significant reduction 4 weeks after the end of treatment. Attention and impulsivity in the computer based attention test TAP improved significantly (Go/NoGo: speed, variability, commissions; Flexibility: variability, commissions). According to these results, NIRS-neurofeedback might be as effective in reducing the main symptoms of ADHD, as it was shown before in randomized controlled studies for EEG-neurofeedback (e.g., Gevensleben et al., 2009; Meisel et al., 2013).

The effect size for the parents’ ratings of ADHD symptoms in the NIRS-group was high for pre-post comparison (*r* = −0.587). This is comparable to effect-sizes of EEG-neurofeedback as reported in the meta-analysis of Arns et al. (2009). Here, high effect sizes for inattention and impulsivity and medium effect sizes for hyperactivity in pre-post designs of EEG-neurofeedback-studies were observed. With larger sample sizes in NIRS-neurofeedback, a differentiated analysis of effects on symptom groups (inattention, impulsivity, hyperactivity) could be conducted, allowing detailed comparisons with effect sizes of EEG-neurofeedback.

The general question of feasibility of NIRS-neurofeedback for children with ADHD can be answered by taking into account different variables. On the one hand, the technical implementation was possible; on the other hand, children and parents accepted the procedure. All nine children took part in all twelve sessions. At the beginning of each training session, motivation was rated on a 4-point smiley scale (1 = totally motivated, 2 = quite motivated, 3 = not much motivated, 4 = not motivated at all). The mean motivation over all sessions and children was high (*M* = 1.51; *sd* = 0.89). Parents rated their satisfaction with the training on a six-item scale 4 weeks and 6 months after the training. They were asked to rate satisfaction with the training, satisfaction with the trainer, empathy of the trainer, trust in the training, trust in the competence of the trainer and recommendation of the training on a 7-point scale (endpoints: 0 = not at all, 7 totally). The mean of parent satisfaction 4 weeks and 6 months after training was high (post test 2: *M* = 5.48, *sd* = 0.58, follow-up test: *M* = 5.57, *sd* = 0.57). Additionally, parents were asked for adverse side effects in relation to measurement and training at all measurement points. No serious adverse events were documented. Two children reported to have had transient headaches directly after some of the training sessions, possibly caused by the fixation of the probe set. In conclusion, NIRS-neurofeedback seems to be a feasible and accepted intervention for children with ADHD.

### Comparison with EEG-neurofeedback and EMG-feedback and future directions

The NIRS-group showed a significant reduction of ADHD symptoms in parents’ ratings after twelve training sessions. A trend towards decreased ADHD symptoms was observed for the EEG- and the EMG-group. In the between group comparison there were no significant differences in symptom reduction. Despite the matching, the three groups differed significantly in some of the initial values of clinical impairment (quality of life and associated behavioral problems).

NIRS-neurofeedback was effective in the reduction of the main symptoms of ADHD and possibly more time-effective in comparison to EEG-neurofeedback. However, this interpretation has to be confirmed, due to the fact that differences in improvement after the 12 sessions did not reach significance, and that the initial values in quality of life and associated behavioral symptoms of the groups differed. Moreover, a completed intervention was compared with two uncompleted interventions, based on the assumption of a more rapid improvement with NIRS neurofeedback, and the sample sizes were small. For future studies, sample sizes have to be enlarged and a randomized controlled design is mandatory. NIRS-neurofeedback could enlarge the treatment options for ADHD, with the possible advantage of being a shorter intervention in comparison to EEG-neurofeedback. The analysis of NIRS-data throughout the training will hopefully allow conclusions as regards to the learning of self-regulation and number of sessions needed. Birbaumer et al. (2013) assume that faster learning of self-regulation of blood oxygenation in comparison to neuroelectricity is associated with the sensoric input processed for the vascular system allowing a faster development of an adequate response. Future studies should analyze the differences in the protocols and learning curves of NIRS- and EEG-neurofeedback to gain more insights into the underlying mechanisms of self-regulation. As regards to the different velocity of the feedback signals one might speculate that a slower signal facilitates learning. In the absence of any study investigating this issue it should be noted for future research.

As a limitation it has to be taken into account, that medication could have a distorting effect on the results: seven of nine children in each group received a medication with methylphenidate during the training. The effects of medication were not assessable in this study. It might be possible, that symptom reduction only occurs in less impaired children or because medication allows a better training. Larger sample sizes, subgroups with medicated and unmedicated children are necessary to control for effects of medication. As an example, results of the multicenter study (comparison of EEG- and EMG-feedback), show that effects of neurofeedback were independent of medication (Holtmann et al., 2014c) and the relationship between symptom severity and outcome is inverse.

Whether NIRS-neurofeedback can be implemented as a stand-alone or part of a multimodal treatment of ADHD will only be answered after studies with a corresponding design. A combination of different interventions according to individual forming of problems is another field of future research in the treatment of ADHD. A multi-center stepped care study dealing with severity-adapted combined interventions including SCP-neurofeedback will be conducted in Germany from February 2015 (ESCAlife: Evidence-based, Stepped Care of ADHS along the life-span)^1^. Results could give a hint on additional effects of neurofeedback and medication.

NIRS-neurofeedback is a promising intervention for children with ADHD and can enlarge the range of options for a treatment of ADHD. Future studies should focus on randomized controlled designs. Especially the comparison with EEG-neurofeedback, and with its final rather than its intermediate outcome, is necessary to further support the assumption that NIRS-neurofeedback needs fewer sessions for comparable symptom reduction. It would also be important to clarify whether longer NIRS-neurofeedback training (i.e., with more than 12 sessions) yields further clinical improvement. For further development of NIRS-neurofeedback the identification of other possible feedback regions based on the growing number of NIRS-studies with children with ADHD is required. The prefrontal cortex plays a central role in ADHD. However, involving a greater database and identifying target regions according to symptomatology could lead to an evidence-based adaption of feedback protocols for individualized treatment of ADHD.

## Conflict of interest statement

Anna-Maria Marx, Adrian Furdea, Yvonne Fuchsenberger, Holger Gevensleben, Daniel Brandeis, Ann-Christine Ehlis and Andreas J. Fallgatter declare no commercial or financial relationships that could be construed as a potential conflict of interest. Ute Strehl was paid for public speaking by Novartis, Medice, Neuroconn, the German Society for Biofeedback and Akademie König und Müller. Martin Holtmann served in an advisory or consultancy role for Lilly, Shire and Bristol-Myers Squibb, and received conference attendance support or was paid for public speaking by Bristol-Myers Squibb, Janssen-Cilag, Lilly, Medice, Neuroconn, Novartis and Shire. Christine M. Freitag received one time speaker’s fees by Ely Lilly and Shire over the last 3 years. Tobias Banaschewski served in an advisory or consultancy role for Hexal Pharma, Lilly, Medice, Novartis, Otsuka, Oxford outcomes, PCM scientific, Shire and Viforpharma. He received conference attendance support and conference support or received speaker’s fee by Lilly, Medice, Novartis and Shire. He is/has been involved in clinical trials conducted by Lilly, Shire and Viforpharma. The present work is unrelated to the above grants and relationships. Prof. Rothenberger is member of an advisory board and speakers’ bureau of Lilly, Shire, Medice and Novartis. He got research and travel support and an educational grant from Shire and research support from the German Research Society. Where applicable, the above mentioned authors declare that the present work is unrelated to the above mentioned grants and relationships.

## Footnotes

^1^Project description: http://akip.uk-koeln.de/forschung-publikation/forschungsprojekte/fg_esca_06_2014_weg.pdf [14.09.2014]
